# Connexin 43 Plays a Role in Pulmonary Vascular Reactivity in Mice

**DOI:** 10.3390/ijms19071891

**Published:** 2018-06-27

**Authors:** Myo Htet, Jane E. Nally, Andrew Shaw, Bradley E. Foote, Patricia E. Martin, Yvonne Dempsie

**Affiliations:** Department of Life Sciences, School of Health and Life Sciences, Glasgow Caledonian University, Glasgow G4 0BA, UK; Myo.Htet@gcu.ac.uk (M.H.); J.E.Nally@gcu.ac.uk (J.E.N.); andrew1994@live.co.uk (A.S.); BFOOTE200@caledonian.ac.uk (B.E.F.); patricia.martin@gcu.ac.uk (P.E.M.)

**Keywords:** pulmonary arterial hypertension (PAH), connexin43 (Cx43), gap junction, vascular reactivity, nitric oxide, serotonin, endothelin-1, isoprenaline

## Abstract

Pulmonary arterial hypertension (PAH) is a chronic condition characterized by vascular remodeling and increased vaso-reactivity. PAH is more common in females than in males (~3:1). Connexin (Cx)43 has been shown to be involved in cellular communication within the pulmonary vasculature. Therefore, we investigated the role of Cx43 in pulmonary vascular reactivity using *Cx43* heterozygous (*Cx43^+/−^*) mice and ^37,43^Gap27, which is a pharmacological inhibitor of Cx37 and Cx43. Contraction and relaxation responses were studied in intra-lobar pulmonary arteries (IPAs) derived from normoxic mice and hypoxic mice using wire myography. IPAs from male *Cx43^+/−^* mice displayed a small but significant increase in the contractile response to endothelin-1 (but not 5-hydroxytryptamine) under both normoxic and hypoxic conditions. There was no difference in the contractile response to endothelin-1 (ET-1) or 5-hydroxytryptamine (5-HT) in IPAs derived from female *Cx43^+/−^*mice compared to wildtype mice. Relaxation responses to methacholine (MCh) were attenuated in IPAs from male and female *Cx43^+/−^* mice or by pre-incubation of IPAs with ^37,43^Gap27. N_ω_-Nitro-L-arginine methyl ester (l-NAME) fully inhibited MCh-induced relaxation. In conclusion, Cx43 is involved in nitric oxide (NO)-induced pulmonary vascular relaxation and plays a gender-specific and agonist-specific role in pulmonary vascular contractility. Therefore, reduced Cx43 signaling may contribute to pulmonary vascular dysfunction.

## 1. Introduction

Pulmonary arterial hypertension (PAH) is a progressive disease in which the mean resting pulmonary artery pressure rises above 25 mmHg with a mean resting capillary wedge pressure lower than 15 mmHg [1]. This increase in pressure is associated with both constriction and remodeling of the distal pulmonary vasculature and eventually leads to right-sided heart failure. Prognosis is poor and survival has been reported to only 68% after three years on therapy [2]. PAH is far more common in females than in males (~3:1) [3]. Dysregulation of cell-to-cell communication particularly between pulmonary artery endothelial cells (PAECs) and pulmonary artery smooth muscle cells (PASMCs) is thought to play an important role in both constriction and remodeling of the pulmonary vasculature in PAH [4]. For example, PAECs from patients with PAH have increased gene and protein expression of tryptophan hydroxylase 1 (Tph1), which is the rate-limiting enzyme in the synthesis of 5-hydroxytryptamine (5-HT) [5]. 5-HT causes contraction of pulmonary arteries and proliferation of PASMCs [6]. In addition, PAECs from PAH patients produce decreased amounts of nitric oxide, which is a potent vasodilator that suppresses proliferation of PASMCs [7].

Connexins are transmembrane proteins that can oligomerize to form a pore in the cell membrane known as a hemi-channel with small regulatory molecules such as 3′,5′-cyclic adenosine monophosphate (cAMP), adenosine 5′ triphosphate (ATP), calcium (Ca^2+^), and inositol 1,4,5-triphosphate (IP_3_), which can pass directly through the cell membrane. Hemi-channels on adjacent cells can align to form gap junctions that allow these mediators to pass directly from the cytoplasm of one cell to that of another. Connexins 37 (Cx37), 40 (Cx40), 43 (Cx43), and 45 (Cx45) are expressed in diverse networks throughout the vasculature [8,9,10]. In the systemic vasculature, connexins have established roles in regulating vascular tone, vascular cell growth, angiogenesis, cell differentiation, and development [11]. Pannexins belong to the same super-family as connexins and also form membrane-associated channels that can mediate the release of small signaling molecules [12]. Pannexin-1 (Panx1) is expressed throughout the vasculature in the endothelium and also in the medial layer of small resistance arteries [13] and has been shown to play an important role in the α-adrenoceptor mediated contractile response [14].

Recent evidence suggests that dysregulated connexin signaling is involved in the pathophysiology of PAH. For example, blood outgrowth endothelial cells from patients with PAH show abnormal gap junctional communication. In addition, the nitric oxide synthase (NOS) inhibitor asymmetric dimethyl arginine (ADMA), is upregulated in PAH patients and inhibits gap junctional communication in human PAECs. The effects of ADMA are prevented by over-expression of Cx43 or by treatment with rotigaptide, which enhances connexin coupling [15]. In line with this, Cx37 and Cx40 are down-regulated in PAECs from PAH patients. Restoration of the transcription factor myocyte enhancer factor 2 leads to increased expression of various transcriptional targets including Cx37 and Cx40. It also rescues pulmonary hypertension in experimental models [16]. Mice genetically deficient in Cx40 are protected against hypoxic-induced pulmonary hypertension. Both genetic knockdown and pharmacological inhibition of Cx40 attenuates hypoxic pulmonary vasoconstriction in the mouse isolated perfused lung [17]. Moreover, contraction to phenylephrine (an α-1 adrenoceptor agonist) in pulmonary arteries taken from chronic hypoxic and monocrotaline (MCT) treated rats is inhibited by both ^37,43^Gap27 and ^40^Gap27 (connexin mimetic peptide blockers of Cxs37/ 43 and Cx40, respectively) [18].

Furthermore, Cx43 has been shown to play a role in 5-HT mediated signaling in the pulmonary vasculature. 5-HT can be synthesized in human PAECs by Tph1 and then released to act on neighboring PASMCs to mediate both proliferation and contraction [6,19,20,21]. Studies have shown in experiments using co-cultures of rat PAECs and rat PASMCs that 5-HT can pass through myoendothelial gap junctions formed principally by Cx43 [22,23]. In line with this, 5-HT-induced contraction of isolated rat pulmonary arteries was attenuated by ^37,43^Gap27 [18]. 

In the current study, we have assessed the role of Cx43 in pulmonary vascular reactivity using pulmonary arteries from *Cx43* heterozygous mice (*Cx43^+/−^* mice). Heterozygous mice were used as a complete genetic knockdown of Cx43, which is lethal in mice [24]. In addition to assessing vasoreactivity in *Cx43^+/−^* mice, we have also assessed vasoreactivity in the presence of ^37,43^Gap27. Since PAH is more common in females than males [3], the role of Cx43 in vascular reactivity was assessed in both male and female mice under normoxic conditions. Paradoxically, male mice have previously been shown to develop more pronounced hypoxic-induced PAH than females [25] and, therefore, our hypoxic experiments were conducted in male mice. Gene expression of *Cx37* (encoded by *GJA4*), *Cx40* (encoded by *GJA5*), *Cx45* (encoded by *GJC1*), and *Panx1* was assessed in pulmonary arteries from *Cx43^+/−^* mice along with gene expression of various mediators known to play a role in the development of PAH: *Tph1*, endothelial nitric oxide synthase (*eNOS*, encoded by NOS3), and bone morphogenetic receptor type II (*BMPRII*, encoded by *BMPR2*). *BMPRII* mutations have been found in patients with various forms of PAH [26]. Dysregulated bone morphogenetic protein (BMP) signaling is thought to be pivotal to the pathophysiology of PAH [27].

## 2. Results

### 2.1. Gene Expression of Connexins in Pulmonary Arteries from Cx43 heterozygous Mice

**Figure 1:**
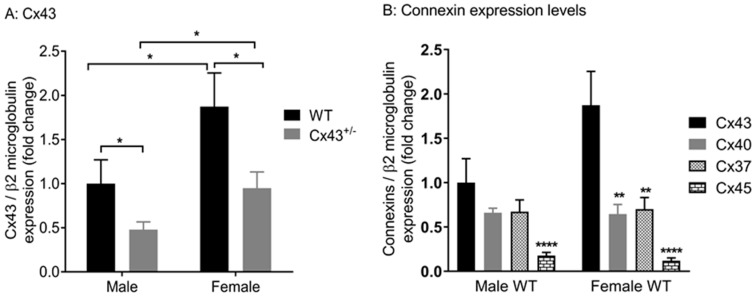
Connexin gene expression in pulmonary arteries from male and female mice. *Cx43* gene expression is reduced in pulmonary arteries from *Cx43* heterozygous (*Cx43^+/−^)* mice (**A**). Female mice have increased levels of *Cx43* compared to males (**A**). *Cx43* is the predominant vascular connexin in female mice while male mice show similar levels of *Cx43*, *Cx40*, and *Cx37*. *Cx45* is expressed in lower levels than *Cxs 43, 40,* and *37* in both male and female mice (**B**). Data are presented as mean ± S.E.M. and were analyzed by two-way ANOVA. *n* = 6 with each sample run in triplicate. **A**: * *p* < 0.05, **B**: ** *p* < 0.01, **** *p* < 0.0001 compared to *Cx43*.

First, quantitative real time PCR (qPCR) was performed to confirm reduced gene expression of Cx43 in pulmonary arteries of male and female *Cx43^+/−^* mice. *Cx43* expression was higher in females than in males in both WT and *Cx43^+/−^* mice (Figure 1A). Afterward, pulmonary arterial gene expression levels of *Cx43* with *Cx37*, *Cx40*, and *Cx45* in wildtype mice were compared. *Cx43* was the predominant vascular connexin in female mice. In male mice, there was a trend towards *Cx43* being expressed at greater levels than *Cx37* and *Cx40*, but this was not significant. *Cx45* was expressed at lower levels in both male and female mice (Figure 1B).

**Figure 2:**
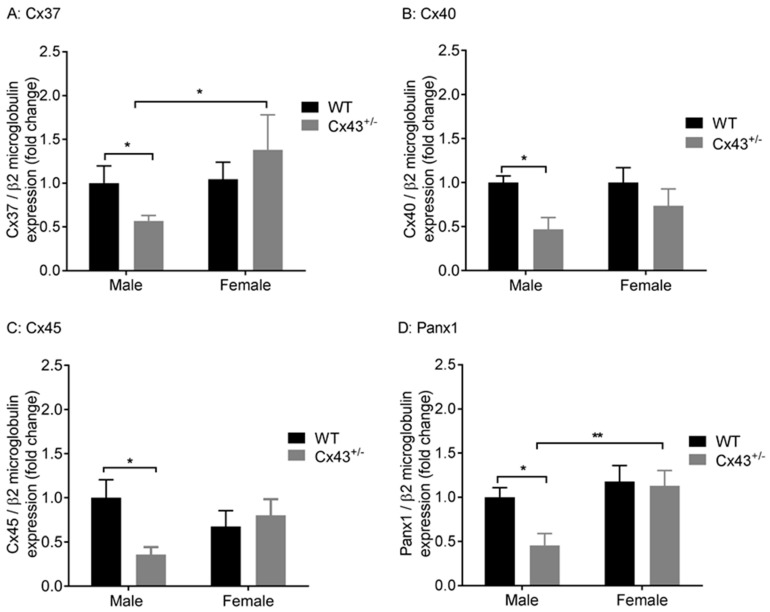
Gene expression of *Cx37* (**A**), *Cx40* (**B**), *Cx45* (**C**), and *Panx1* (**D**) in pulmonary arteries from male and female wildtype (WT) and *Cx43* heterozygous (*Cx43^+/−^)* mice. Data are presented as mean ± S.E.M. and were analyzed by two-way ANOVA. * *p <* 0.05, ** *p <* 0.01, *n* = 6 per group with each sample analyzed in triplicate.

We explored the effects of genetic knockdown of *Cx43* on gene expression of *Cx37, Cx40, Cx45,* and *Panx1*, which are all expressed in the vasculature. Male *Cx43^+/−^* mice displayed reduced gene expression of *Cx37, Cx40, Cx45*, and *Panx1* mRNA (Figure 2A–D) when compared to WT littermates. Female *Cx43^+/−^* mice, however, displayed no changes in gene expression of *Cx37, Cx40, Cx45,* or *Panx1* compared to WT littermates (Figure 2A–D). The housekeeping gene *β2-microglobulin* was expressed at similar levels in all four groups of mice studied (*C*t values: male WT 24.9 ± 0.2, male *Cx43^+/−^* 24.4 ± 0.2, female WT 25.0 ± 0.2, female *Cx43^+/−^* 24.6 ± 0.3, *p* = 0.86).

### 2.2. Pulmonary Arterial Contractile Responses

**Figure 3:**
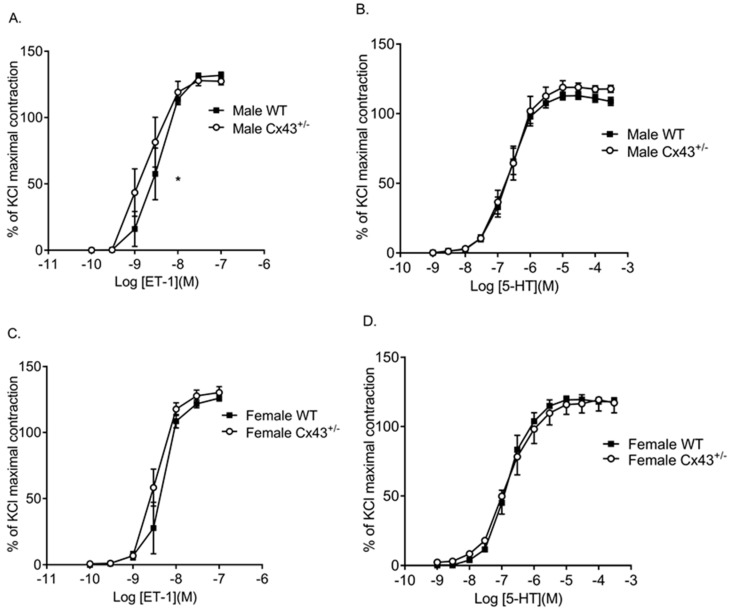
Pulmonary vascular contractility to ET-1 and 5-HT in intralobar pulmonary arteries (IPAs) from male and female wildtype (WT) and *Cx43* heterozygous (*Cx43^+/−^*) mice. ET-1 was more potent in IPAs from male *Cx43^+/−^* mice than WT mice (**A**). There was no difference in contractile response to 5-HT in IPAs from male WT and *Cx43^+/−^* mice (**B**). There was no difference in ET-1 (**C**) or 5-HT (**D**) induced contractile response in IPAs from female WT or *Cx43^+/−^* mice. Data are shown as mean ± S.E.M. Global differences in concentration response curves were compared by two-way ANOVA. Changes in logarithm of median effective concentration (Log EC_50_) and maximal contractile responses (E_max_) between two different groups were analyzed by using the Student’s *t*-test. * EC_50_ is significantly (*p* < 0.05) reduced in male *Cx43^+/−^* mice, *n* = 5–7 per group.

In the intra-lobar pulmonary arteries (IPAs) of male mice, endothelin-1 (ET-1) was more potent (had a lower median effective concentration or EC_50_ value) in *Cx43^+/−^* mice compared to WT mice (Figure 3A; Table 1). Maximal response to ET-1 (E_max_) was, however, unchanged between WT and *Cx43^+/−^* mice. There was no global shift in the concentration response curve (Figure 3A; Table 1). IPAs from male *Cx43^+/−^* mice showed similar contractile responses to 5-hydroxytryptamine (5-HT) as those from WT mice (Figure 3B; Table 1). Contractile responses to both ET-1 and 5-HT were similar in IPAs from female *Cx43^+/−^* mice compared to female WT mice (Figure 3C,D; Table 1). 

### 2.3. Pulmonary Arterial Relaxation Responses

**Figure 4:**
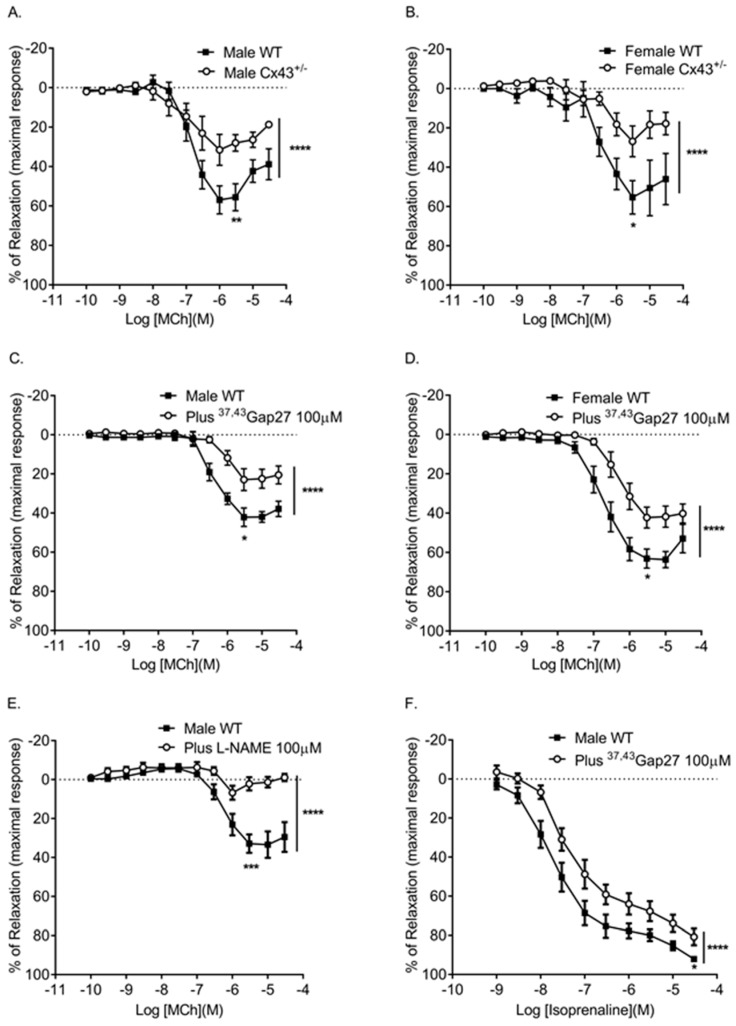

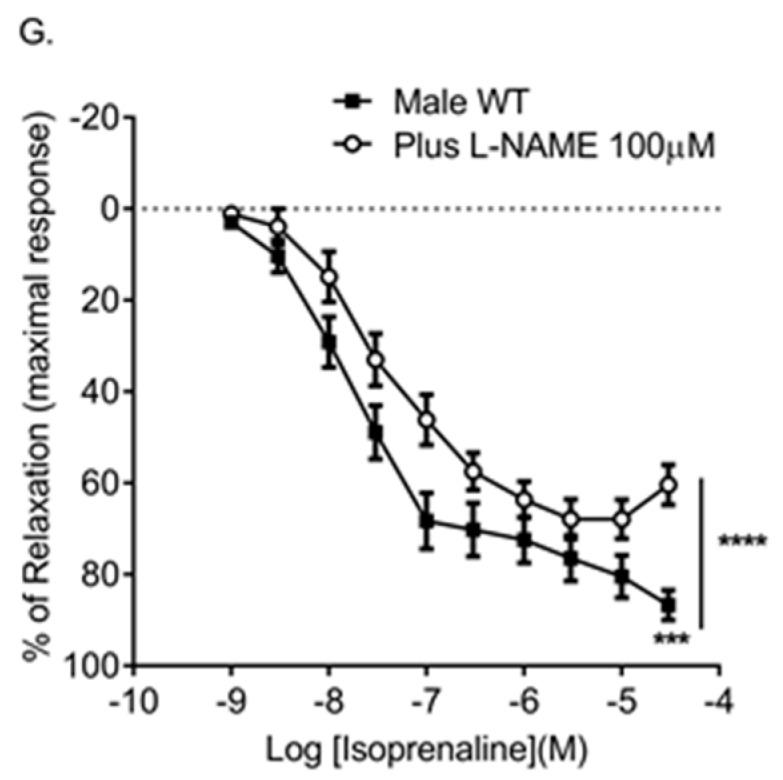
Effects of genetic reduction or pharmacological inhibition of Cx43 on pulmonary vascular relaxation responses. Male (**A**) and female (**B**) *Cx43* heterozygous (*Cx43^+/−^*) mice show reduced relaxation in response to MCh. Pre-incubation with ^37,43^Gap27 also reduced the relaxation response in male (**C**) and female (**D**) mice. MCh-induced relaxation was ablated in the presence of l-NAME (**E**). ^37,43^Gap27 partially inhibited isoprenaline-induced relaxation in IPAs from male mice (**F**) as did l-NAME (**G**). Global differences in concentration response curves were compared by two-way ANOVA. Changes in logarithm of median effective concentration (Log EC_50_) and maximal relaxation responses (R_max_) between two different groups were analyzed by using the Student’s *t*-test. Data are shown as mean ± S.E.M. * *p* < 0.05, ** *p* < 0.01, *** *p* < 0.001, **** *p* < 0.0001, *n =* 5–6 per group. Statistical symbols shown on the right hand side of graphs indicate global shifts in the concentration response curves. Statistical symbols underneath curves indicate changes in maximal relaxation (R_max_) values.

The relaxation response produced by methacholine (MCh) was significantly reduced in IPAs of both male and female *Cx43^+/−^* mice when compared to WT mice (Figure 4A,B; Table 2). Pharmacological inhibition of Cx43 with ^37,43^Gap27 also significantly attenuated the relaxation responses produced by MCh in IPAs of both male and female mice (Figure 4C,D; Table 2). 

We then confirmed that MCh-induced relaxation responses were dependent upon nitric oxide (NO) since the l-NAME completely inhibited MCh-induced relaxation (Figure 4E; Table 2). Then we assessed the role of Cx43 in isoprenaline-induced relaxation. Isoprenaline is classically thought to induce relaxation via the cAMP pathway. In these experiments, the relaxation induced by isoprenaline was partially inhibited by ^37,43^Gap27 (Figure 4F; Table 2). Furthermore, we showed nitric oxide plays a role in isoprenaline-induced relaxation since the l-NAME partially attenuated isoprenaline-induced relaxation (Figure 4G; Table 2).

### 2.4. Gene Expression of Bone Morphogenetic Protein Receptor Type II, Tryptophan Hydroxylase 1, and Endothelial Nitric Oxide Synthase in Pulmonary Arteries from Cx43 Heterozygous Mice

**Figure 5:**
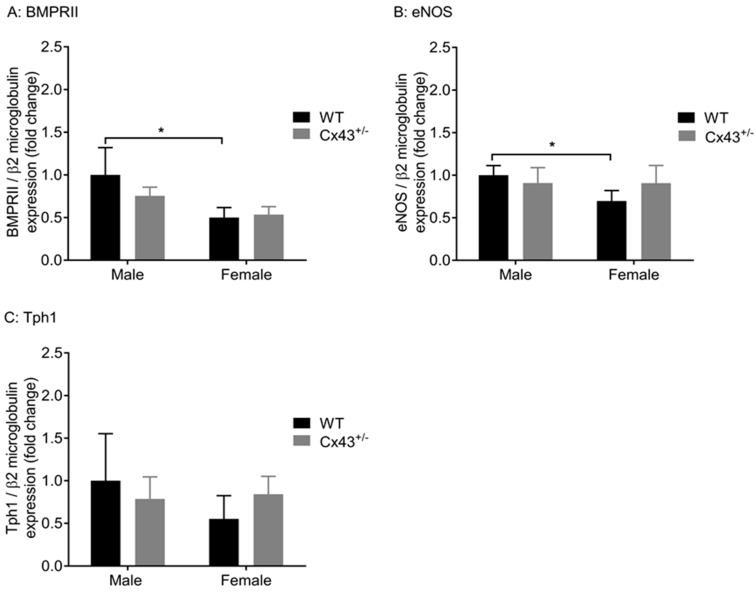
Gene expression of BMPRII (encoded by *BMPR2*) (**A**), eNOS (encoded by *NOS3*) (**B**), and *Tph1* (**C**) in pulmonary arteries of male and female WT and *Cx43^+/−^* mice. No differences were observed in expression of *BMPR2*, *NOS3*, or *Tph1* between WT and Cx43^+/−^ mice (either male or female). *BMPR2* and *NOS3* were significantly downregulated in female WT mice compared to male WTs. Data are presented as mean ± S.E.M. and were analysed by using two-way ANOVA. * *p* < 0.05, *n* = 6 per group with each sample run in triplicate.

Since *Cx43^+/−^* mice displayed dysregulated pulmonary vascular reactivity, gene expression of *BMPRII* (encoded by *BMPR2*), *eNOS* (encoded by *NOS3*), and *Tph-1*, mediatorsimportant for regulating pulmonary vascular function, were assessed. Expression of *BMPR2, NOS3* , and *Tph-1* were not significantly altered in either male or female *Cx43^+/−^* mice compared to WT littermates (Figure 5A–C). The expression of *NOS3* and *BMPR2* were significantly lower in female WT mice when compared to male WT mice (Figure 5A,B). 

### 2.5. Effects of Hypoxia on Pulmonary Vascular Contractility in Male Cx43 Heterozugous Mice

**Figure 6:**
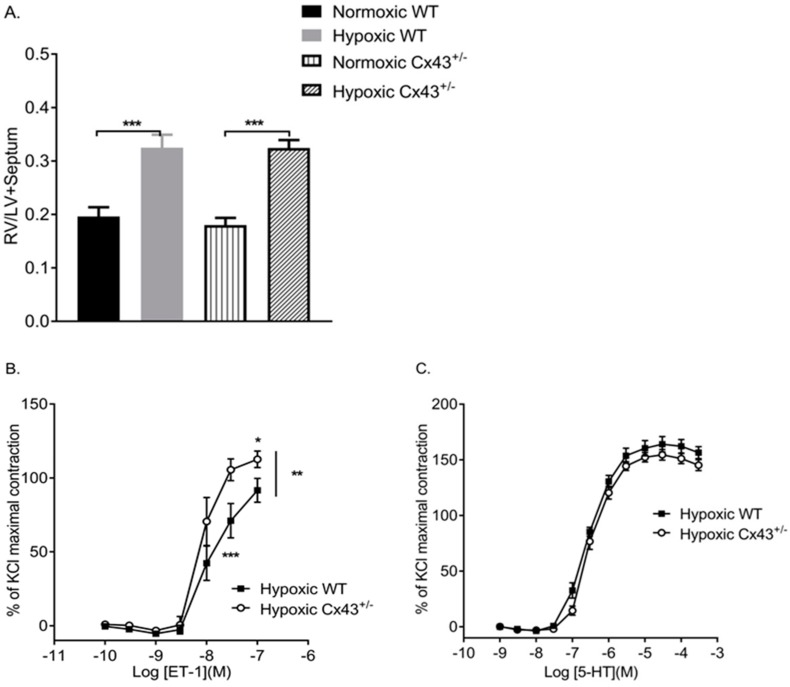
Assessment of the development of right ventricular hypertrophy by right ventricular weight ratio left ventricle plus septal weight (RV/LV+Septum) (**A**). Concentration response curves (CRCs) to ET-1 (**B**) and 5-HT (**C**) in intra-lobar pulmonary arteries derived from hypoxic wildtype (WT) and hypoxic *Cx43* heterozygous (*Cx43^+/−^)* mice. Data are shown as mean ± S.E.M. Data in panel **A** were analyzed by two-way ANOVA. In panels **B** and **C**, global differences in CRCs were compared by two-way ANOVA. Changes in the logarithm of median effective concentration (Log EC_50_) and maximal contractile responses (E_max_) between two different groups were analyzed by using the Student’s *t*-test * *p <* 0.05, ** *p* < 0.01, *** *p <* 0.001, *n =* 5–7 per group. The statistical symbol shown on the right hand side of graph B indicates a global shift in the CRC. The symbol underneath the curve indicates changes in the median effective concentration (EC_50_) while the symbol above the curve indicates changes in the maximal response (E_max_).

The effects of chronic hypoxia on pulmonary vascular reactivity in *Cx43^+/−^* mice were investigated. The hypoxic experiments were carried out in male mice since it has been previously shown that male mice are more susceptible to hypoxic-induced PH than female mice [25]. Both WT and *Cx43^+/−^* mice developed the right ventricular hypertrophy after two weeks of chronic hypoxic exposure, which verifies the mouse hypoxic model (Figure 6A). IPAs derived from chronic hypoxic *Cx43^+/−^* mice showed an increased sensitivity to ET-1, which was assessed by a reduced EC_50_ value and a global leftward shift in the contractile response. In addition, the maximal contractile effect produced by ET-1 was significantly greater in IPAs from hypoxic *Cx43^+/−^* mice than from hypoxic WT mice (Figure 6B; Table 3). There was no difference in a contractile response to 5-HT in IPAs from hypoxic *Cx43^+/−^* mice (Figure 6C; Table 3).

### 2.6. Effects of Hypoxia on Expression of Cx43 in Mouse Lung and Pulmonary Artery 

**Figure 7:**
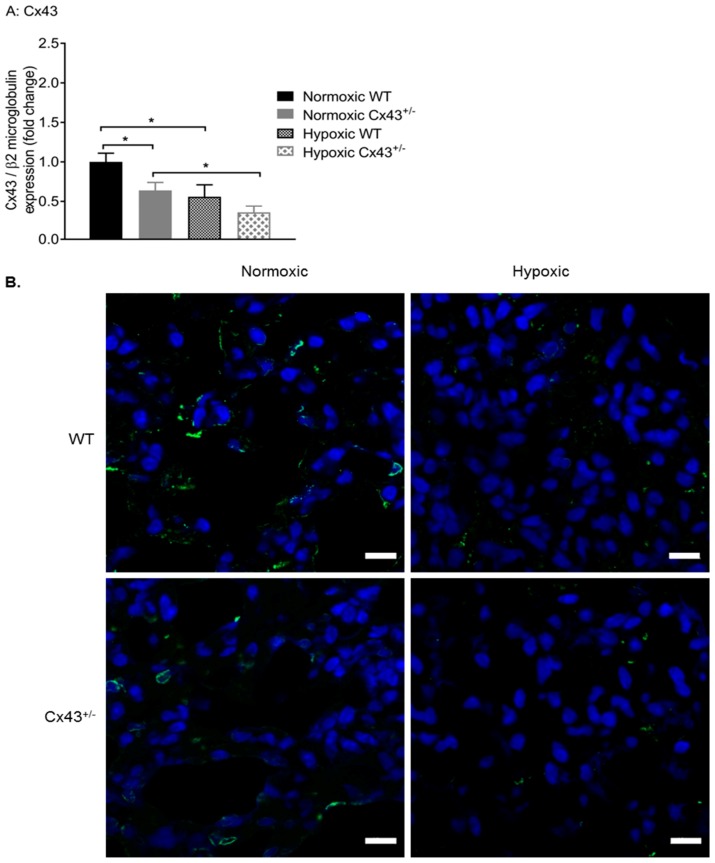
Cx43 expression in pulmonary arteries of wildtype (WT) and *Cx43* heterozygous (*Cx43^+/−^)* mice under normoxic and chronic hypoxic conditions. *Cx43* gene expression is reduced by hypoxia in both WT and *Cx43^+/−^* mice (**A**). Confocal images of Cx43 immunofluorescence staining in normoxic and hypoxic WT and *Cx43^+/−^* mouse lung tissue sections are shown in (**B**). Green fluorescence punctate findings represent Cx43 immunoreactivity and blue staining represents nuclei. Scale bars represent 10 µm. For panel **A**, data is presented as mean ± S.E.M. and was analyzed by two-way ANOVA, * *p <* 0.05, *n =* 6 with each sample run in triplicate.

Afterward, the effects of chronic hypoxia on Cx43 gene and protein expression were assessed. Using qPCR, it was shown that hypoxia significantly down-regulated *Cx43* gene expression in both WT and *Cx43^+/−^* male mice (Figure 7A). We then used immunofluorescence to visualize the effects of hypoxia on Cx43 protein expression. Lung expression of Cx43 (green fluorescence) was reduced in hypoxic WT mice when compared to normoxic WT mice (Figure 7B). Furthermore, Cx43 immunoreactivity was further reduced in lungs of hypoxic *Cx43^+/−^* mice when compared to normoxic *Cx43^+/−^* mice (Figure 7B).

### 2.7. Effects of Hypoxia on Gene Expression in Pulmonary Arteries Derived from WT and Cx43^+/−^ Mice

**Figure 8:**
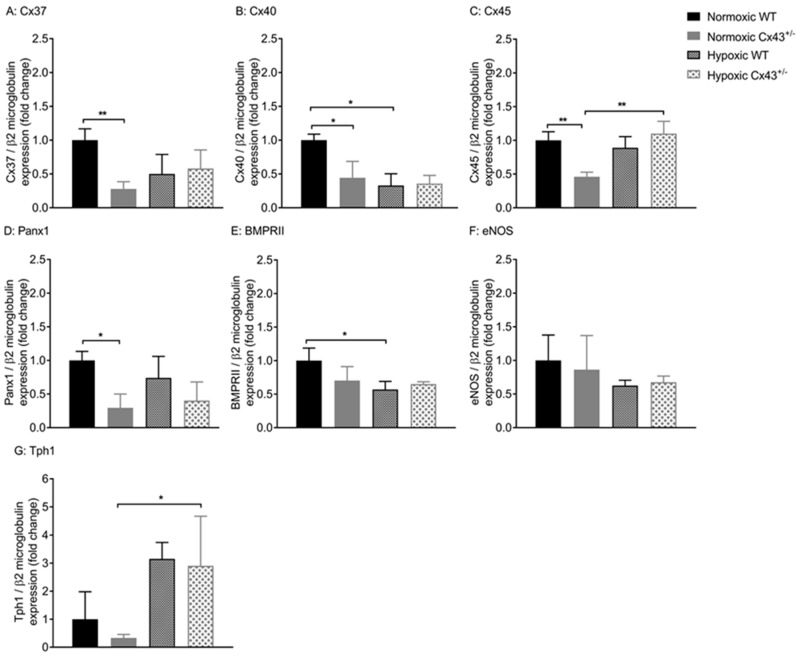
Gene expression of *Cx37* (**A**), *Cx40* (**B**), *Cx45* (**C**), *Panx1* (**D**), *BMPRII* (**E**), *eNOS* (**F**) and *Tph1* (**G**) in pulmonary arteries of wildtype (WT) and *Cx43* heterozygous (*Cx43^+/−^)* mice under normoxic and chronic hypoxic conditions. All data are presented as mean ± S.E.M. and were analyzed by two-way ANOVA. * *p <* 0.05, ** *p <* 0.01, *n =* 6 per group with each sample run in triplicate.

As chronic hypoxia mediated increased vascular reactivity to ET-1 and decreased Cx43 expression, the effects of chronic hypoxia on the expression of other vascular connexins and *Panx1* were assessed (Figure 8 A–D). Hypoxia mediated a downregulation of *Cx40* gene expression in WT mice (Figure 8B) while gene expression of *Cx45* was up-regulated by hypoxia in *Cx43^+/−^* mice (Figure 8C). Hypoxia had no effect on the expression of *Cx37* or *Panx1* in WT or *Cx43^+/−^* mice (Figure 8A,D). 

Gene expression of *BMPR2*, *Tph1*, and *NOS3* in response to hypoxia in *Cx43^+/−^* mice was also assessed. *BMPR2* was downregulated by hypoxia in WT mice and *Tph1* was up-regulated by hypoxia in *Cx43^+/−^* mice. There were no differences in *BMPR2*, *NOS3*, or *Tph1* expression between hypoxic WT and hypoxic *Cx43^+/−^* mice (Figure 8E–G).

## 3. Discussion

To our knowledge, this is the first study to show that mice genetically deficient in *Cx43* develop pulmonary vascular dysfunction. It is also the first study to show that Cx43 plays a role in NO-induced pulmonary vascular relaxation. These findings add to the growing body of evidence that suggests that Cx43 is involved in regulation of the pulmonary vasculature. Since PAH is associated with altered pulmonary vascular reactivity, these data suggest Cx43 is worthy of further investigation as a novel therapeutic target for PAH.

Relaxation responses to MCh were reduced in IPAs from both male and female *Cx43^+/−^* mice. Subsequently, we found that pharmacological inhibition of Cx43 using ^37,43^Gap27 also attenuated MCh-induced relaxation. ^37,43^Gap 27 inhibits both Cx37 and Cx43 and both Cx37 and Cx43 are down-regulated in male *Cx43^+/−^* mice. However, the vascular connexins of only Cx43 are downregulated in female *Cx43^+/−^* mice. Therefore, reduction of MCh-induced relaxation in female *Cx43^+/−^* mice suggests an important role for Cx43 in this effect. Our studies confirmed that MCh-induced relaxation was NO-dependent since the MCh mediated relaxation responses were completely abolished in the presence of l-NAME. It is widely considered that NO can diffuse through the endothelial and smooth muscle cell membranes to activate guanylate cyclase within the smooth muscle cell and mediate vasodilation. It has been shown, however, that diffusion of NO across the vascular cell membrane requires specific connexin channels [28]. NO opened and permeated hemichannels expressed in HeLa cells transfected and selected to express Cx43, Cx40, or Cx37. In addition, the blockade of connexin channels abolished myoendothelial NO transfer and NO-dependent vasodilation induced by acetylcholine in rat mesenteric arteries [28]. There is also mounting evidence that NO can interact with gap junctions in a complex and inter-dependent fashion [29]. Cx43 is constitutively S-nitrosylated at cysteine 271 by NO at the myoendothelial gap junctions. This nitrosylation keeps the myoendothelial gap junction open and denitrosylation closes the gap junction channel [30]. Additionally, NO has been shown to enhance gap junction coupling and increase trafficking of Cx40 to the membrane in endothelial cells via the protein kinase A activation [31]. Cx40 has been shown to co-localize with eNOS in the mouse aorta and is involved in conducting vasodilation [32]. *Cx40* knock out (*Cx40^−/−^*) mice showed reduced basal and acetycholine induced NO release and reduced eNOS expression in aortas [32]. However, NO can act as a negative regulator of Cx37, which reduces Cx37-mediated dye transfer and electrical coupling in human umbilical vascular endothelial cells (HUVEC) and mouse microvascular endothelial cells [33,34]. Therefore, it is possible that Cx43 and NO interact in such a fashion that when Cx43 is genetically downregulated or pharmacologically inhibited, it leads to a reduction in NO-mediated vasorelaxation. In the present study, pulmonary arterial gene expression of *eNOS* was unchanged in male and female *Cx43^+/−^* mice when compared to WT controls. It would, however, be of interest to investigate eNOS protein levels and also MCh-induced NO release in pulmonary arteries from WT and *Cx43^+/−^* mice. It would also be of interest to investigate potential interactions between eNOS and Cx43 using co-immunoprecipitation. 

^37,43^Gap27 also mediated a small but significant inhibition of isoprenaline-induced relaxation. Isoprenaline is classically thought to act through the cAMP pathway. In the present study, isoprenaline-induced relaxation was, however, partially inhibited by l-NAME, which suggests that NO plays a role in isoprenaline-induced relaxation. A previous study has also shown β_2_ adrenoceptor induced relaxation in mouse pulmonary arteries are attenuated by the l-NAME, endothelial denudation, or deletion of *eNOS* [35]. β_2_ adrenoceptors have been detected in the mouse pulmonary endothelial layer by immunostaining [35]. The inhibitions of isoprenaline relaxation mediated by ^37,43^Gap27 and l-NAME were of a similar magnitude. It is, therefore, possible that inhibition of connexin mediated communication via ^37,43^Gap27 inhibits the NO component of isoprenaline relaxation. In future studies, it would be of interest to analyze whether smooth muscle responses to NO are affected by downregulation of Cx43. This could be achieved using an NO donor such as sodium nitroprusside. 

Enhanced ET-1 mediated contraction was observed in IPAs derived from both normoxic and hypoxic male *Cx43^+/−^* mice compared to their WT counterparts. Normoxic female *Cx43^+/−^* mice did not show an increased contractile response to ET-1. However, male *Cx43^+/−^* mice showed downregulation of *Cx37, Cx40, Cx45,* and *Panx1*. These effects were not observed in female *Cx43^+/−^* mice. Downregulation of *Cx37, Cx40, Cx45,* or *Panx1* may, therefore, play a role in the increased contractile response to ET-1 observed in the male *Cx43^+/−^* mice. The reduced Cx43 expression observed in the pulmonary arteries of male mice compared to female mice observed in this study may also contribute to the increased effects of ET-1 in male *Cx43^+/−^* mice. Gender differences in the endothelin system may also contribute to contractile differences between male and female *Cx43^+/−^* mice [36]. Studies have shown that plasma endothelin levels were higher in men when compared to women [37,38]. At the receptor level, the ratio of ET_A_ and ET_B_ receptors in the endothelium of saphenous vein was greater in male subjects (3:1) than in female subjects (1:1) [39]. Haemodynamically, a rat model showed pressor responses induced by intravenous administration of ET-1 were greater in male rats than in female rats [40]. In vitro cell culture studies have also confirmed that 17-β oestradiol (E2) inhibited ET-1 gene expression through the extracellular signal regulated kinase (ERK) pathway [41].

5-HT mediated contractile responses in the IPAs were not affected by the partial loss of *Cx43* in either male or female mice under either normoxic or hypoxic conditions. Therefore, the effects of reduced *Cx43* gene expression varied according to the agonist used. These results agree with previously published data showing that the effects of pharmacological inhibition by ^37,43^Gap27 on contractile responses in IPAs were dependent on the agonist used [18]. In line with the current results, Billaud et al. showed that ^37,43^Gap27 had no effect on 5-HT induced contraction in IPAs derived from hypoxic rats. However, they did report that ^37,43^Gap27 could inhibit 5-HT induced contraction in IPAs from normoxic rats. In contrast with the results reported here showing that downregulation of *Cx43* enhanced ET-1 mediated contraction in IPAs form both normoxic and hypoxic *Cx43^+/−^* mice, Billaud et al. found that ^37,43^Gap27 had no effect on ET-1 induced contraction in IPAs derived from normoxic or hypoxic rats [18]. 5-HT and ET-1 have different mechanisms of contraction. Since connexins are modulated by PKA, PKC, and calcium [42], the role of connexin mediated communication in the contractile response differs according to the agonist used. A possible reason for the disparity between the Billaud study and our own could be due to the ratiometric changes in the balance of Cx43:Cx40:Cx37 expression between rats and mice. A number of studies have reported tissue and species specific variation in connexin expression profiles and ^37,43^Gap27 is defined as being specific to the SRPTEKTIFII sequence—conserved between Cx43 and Cx37 but different in Cx40 [43,44,45,46]. In addition, *Cx43^+/−^* mice will have compensatory changes in other genes, which may affect the contractile response while ^37,43^Gap27 inhibits Cx37 as well as Cx43. This also potentially affects the contractile response. 

In the gene expression studies, *Cx43* expression was higher in female mice than in male mice. This is in keeping with a report which showed that Cx43 expression was higher in rat cardiomyocytes derived from females than males [47]. Furthermore, treatment with oestradiol increased Cx43 expression in human myometrium [48]. In addition to this, there is direct evidence that estrogen can regulate *Cx43* gene expression since the promotor region of *Cx43* gene contains an estrogen response element [49].

Chronic hypoxic rodents are a commonly used model for PAH. In the current study, the chronic hypoxia suppressed Cx43 gene and protein expression are located in the pulmonary arteries of both WT and *Cx43^+/−^* mice. Hypoxia can regulate Cx43 expression post-translationally by phosphorylation [50]. For example, one study showed exposure to hypoxia is associated with an increase in phosphorylation of Cx43- serine 368 (Ser368) in human microvascular endothelial cells and this phosphorylation was associated with downregulation Cx43 protein expression [51]. As hypoxia downregulates Cx43 expression, it would be of interest in future studies to assess the effects of hypoxia on MCh-induced relaxation in *Cx43^+/−^* mice. The current study showed *Cx40* expression was also downregulated in hypoxic WT mice. Previous studies have shown that Cx40 expression was reduced during PAH in rats and treatment with sildenafil increased Cx40 expression via BMP signaling [52,53]. We found *Cx45* expression to be upregulated in the *Cx43^+/−^* mice under chronic hypoxic conditions. The function of Cx45 in the vasculature remains unknown even though it has long been known to be expressed in vascular smooth muscle cells [54].

It is interesting to note that *Cx43^+/−^* mice did not develop increased right ventricular hypertrophy (RVH) in response to hypoxia compared to their wildtype counterparts. It has previously been shown by ourselves and others that changes in pulmonary vascular pressures and pulmonary vascular remodeling do not always lead to the development of RVH in mice. For example, increased pulmonary pressures and remodeling have been observed in the absence of RVH in mice that over-express the serotonin transporter, mice that over-express Mts 1, and mice that are dosed with dexfenfluramine [55,56,57]. In addition, Cx43 is highly expressed in cardiac myocytes and is thought to play an important role in hypertrophy of these cells. Expression and localization of Cx43 in cardiac myocytes has been shown to be dynamically regulated in various animal models of cardiac hypertrophy [58]. Therefore, it is possible that cardiac myocytes from Cx43^+/−^ mice are functionally abnormal and, therefore, have an atypical hypertrophic response to hypoxia.

Female *Cx43^+/−^* mice did not exhibit the compensatory reduction in gene expression of *Cx37, Cx40, Cx45,* or *Panx1* that was observed in male *Cx43^+/−^* mice. Multiple lines of evidence show that *Cx43, Cx40*, and *Cx37* are interdependent on each other and compensatory changes occur upon connexin deletion. For instance, in *Cx40* knock out (*Cx40^−/−^)* mice both total and smooth muscle Cx43 protein expression was reduced in the mouse aortas [59]. In addition, in *Cx40^−/−^* mice, the pericellular component of Cx43 staining was lost and there was increased redistribution of Cx43 in the perinuclear region [59]. This suggests deletion of *Cx40* not only leads to Cx43 downregulation but also affects its trafficking. Conversely, another group found that *Cx40^−/−^* mice showed upregulation of Cx37 and Cx43 in the aortic endothelium [60]. In *Cx40^−/−^* neonatal mice, Cx37 protein expression was downregulated in the endothelium and there was an increased Cx37 and Cx43 in the medial layer [61]. Genetic deletion of *Cx37* in mice showed reduction in endothelial Cx40 [61]. In the present study, we have assessed changes in gene expression in the whole pulmonary artery. It would be of interest to assess cell type specific changes in future studies.

Gene expression of *eNOS, BMPRII*, or *Tph1* were not affected by the loss of *Cx43* under normoxic or hypoxic conditions. Among the normoxic WT mice, *eNOS* and *BMPRII* expression was reduced in the females compared to male WT mice. The literature on eNOS expression in PAH patients is contradictory. eNOS was initially reported to be decreased in lungs of PAH patients [62], but evidence later found eNOS expression was unchanged or even increased in PAH patients [63,64]. Furthermore, NO levels have been shown to be reduced in PAH patients [65] and it has been reported that the activity of eNOS rather than its expression was altered in a murine model of PAH [63]. Our findings show that *BMPRII* expression is reduced in pulmonary arteries from female mice, which is in line with proof that activation of the estrogen response element in the promoter region of *BMPRII* can downregulate *BMPRII* expression [66].

In conclusion, this study has shown that Cx43 plays a role in NO-dependent vasodilation in the pulmonary vasculature. Cx43 is also involved in pulmonary vascular contractility. However, effects on contractility are gender-dependent and agonist-dependent. Hypoxia has been shown to decrease Cx43 expression in mouse pulmonary arteries, which is an effect that may contribute to the increased vasoreactivity observed under hypoxic conditions.

## 4. Materials and Methods 

### 4.1. Ethical Statement 

All experimental procedures were carried out in accordance with the United Kingdom Animal Procedures Act (1986) and with the “Guide for the Care and Use of Laboratory Animals” published by the US National Institutes of Health (NIH publication no.85, eighth edition). Ethical approval was granted by the Glasgow Caledonian University Animal Welfare and Ethics Committee (PPL70/7875, 16 September 2013).

### 4.2. Animals

Male and female wild-type (WT) and *Cx43* heterozygous (*Cx43^+/−^*) mice (C57BL6, 5 to 9 months old) were used in this study. The generation of *Cx43^+/−^* mice was originally carried out by replacing exon 2 of the *Cx43* gene with the neomycin resistance gene, which was previously described [24]. Mice were grouped under standard laboratory conditions. All mice had access to a commercial diet and water ad libitum.

### 4.3. Genotyping 

DNA was extracted from ear notch tissues derived from WT and *Cx43^+/−^* mice. Tissues were suspended in 300 µL TNES buffer [10 mM tris(hydroxymethyl)aminomethane (Tris), 0.4 M sodium chloride (NaCl), 100 mM ethylenediaminetetraacetic acid (EDTA), and 0.6% sodium dodecyl sulphate (SDS)] to which 1.5 µL proteinase K (Fisher Scientific, Loughborough, UK) was added. Samples were then incubated overnight at 55 °C. The next day, 84 µL of 5 M NaCl was added to each sample and samples were centrifuged for 10 min. The supernatant was collected and transferred to fresh tubes. DNA was precipitated by adding 200 µL ice cold 100% ethanol to each tube and vortexing. Samples were then centrifuged for 10 min, the supernatant was discarded, and the pellet was retained. Excess salt was removed by adding 200 µL ice cold 75% ethanol and samples were centrifuged again for 10 min. The supernatant was decanted gently and the pellet was allowed to air dry. The pellet was then re-suspended in 15 µL nuclease-free water and stored at −20 °C until use. A polymerase chain reaction (PCR) was then carried out to amplify the *Cx43* (*GJA1*) and neomycin resistance (*neo^r^*) genes. The primers used were as follows: *neo^r^* forward 5′-GATCGGCCATTGAACAAGATG, melting temperature (Tm) = 56.4 °C, molecular weight (MW) = 6808.5, *neo^r^* reverse: 5′-CCTGATGCTCTTCGTCCAGAT Tm = 57.2 °C, MW = 6637.3, *Cx43* forward: 5′-CAGTCTGCCTTTCGCTGT, Tm = 56 °C, MW = 5433; *Cx43* reverse: 5′-GTAGACCGCACTCAGGCT, Tm = 58 °C, MW = 5485. All primers were purchased from Integrated DNA technologies, Belgium. PCR reactions were performed in a thermal cycler (MJ Research PTC-100 Thermal Cycler, Watertown, MA, USA) and comprised an initial denaturation at 95 °C for 3 min followed by 40 cycles of: denaturation at 95 °C for 30 s, annealing at 55 °C for 30 s, and extension at 72 °C for 1 min. A final extension step was carried out at 72 °C for 15 min. Samples were then run in 2% agarose gel (*v*/*v*) for 45 min at 100 V.

### 4.4. Induction of Hypoxia

For induction of hypoxia, male WT and *Cx43^+/−^* mice were placed in a hypobaric chamber for 14 days, which is previously described [19]. The pressure was adjusted to 550 mbar (equivalent to 10% *v*/*v* O_2_) slowly over two days to allow mice to acclimate. The temperature was maintained at 20–22 °C. Control littermates were kept in a normoxic environment.

### 4.5. Tissue Preparation 

Mice were euthanized by injection of phenobarbitone (60 mg/kg i.p.). After death was confirmed, the chest walls were opened using the mediastinal approach and the hearts and lungs were dissected freely.

### 4.6. Wire Myography Studies

Pharmacological experiments were carried out in third generation intra-lobar pulmonary arteries (IPAs; ~300 µm internal diameter), which was previously described [67,68]. IPAs were mounted on a wire myograph (Danish Myo Technology, DMT) in freshly prepared Krebs-Henseleit Solution (composition (mmol/L) NaCl 119, KCl 4.7, CaCl_2_ 2.5, MgSO_4_ 1.2, NaHCO_3_ 25, KH_2_PO_4_ 1.2, and d-glucose 5.5) at (37 °C) and gassed with 95%O_2_ / 5% CO_2_. All chemicals required for Krebs-Henseleit solution were purchased from Fisher Scientific except CaCl_2_ which was purchased from VWR International Ltd. (Lutterworth, Leicestershire, UK). Following equilibration for one hour, IPAs from normoxic mice were placed under pressures of 12–15 mmHg and IPAs from hypoxic mice that were placed under pressures of 30–35 mmHg to mimic the in vivo environment described previously [67]. Arteries were initially constricted with potassium chloride (KCl, 60 mM), which were then washed out. These processes were repeated two times before contractile or relaxation experiments were carried out. For contractile experiments, cumulative concentration response curves (CCRCs) to 5-HT (1 nM–300 µM) or ET-1 (0.1 nM–0.1 µM) were constructed. For relaxation experiments, vessels were pre-constricted with phenylephrine (PE; 3 µM) and CRCs to MCh (0.1 nM–30 µM) or isoprenaline (1 nM–30 µM) were constructed. In a subset of relaxation experiments, vessels were incubated with ^37,43^Gap27 (100 µM) for 30 min prior to MCh or isoprenaline-induced relaxation responses. ^37,43^Gap27 has previously been shown to have an IC_50_ of 31.5 ± 4.1 µM and to produce a maximum effect at 100 µM [69]. We previously used ^37,43^Gap27 at 100 µM [70,71,72]. In another subset of relaxation experiments, the l-NAME (100 µM) was used to inhibit eNOS. In these experiments the l-NAME was applied for 30 min prior to pre-constriction with PE and the concentration of PE was reduced to 30 nM. It should be noted that 30 nM PE in the presence of l-NAME produced a similar contractile response to 3 µM PE alone. Changes in isometric tension were recorded on LabChart 7 software (AD Instruments Pty Ltd., Bella Vista, New South Wales, Australia). PE, L-NAME, MCh, ET and 5-HT were purchased from Sigma-Aldrich Company Ltd. (Gillingham, Dorset, UK).

### 4.7. Quantitative Real Time PCR (qPCR)

Main and branch pulmonary arteries (1st and 2nd order) were homogenized and RNA was extracted using the Nucleospin RNA kit (MACHEREY-NAGEL GmbH & Co. KG, Düren, Germany) as per the manufacturer instructions. RNA was then reverse transcribed to cDNA using the Precision nanoScript^TM^ 2 Reverse Transcription Kit (Primerdesign Ltd., Chandler’s Ford, UK), according to the manufacturer’s protocols. qPCR was performed using Precision PLUS 2x qPCR master mix (Primerdesign Ltd.) II with taqman primers described in Table 4. β_2_-microglobulin (B2M) (assay ID: HK-DD-mo-300, Primerdesign Ltd.) was used as the endogenous control and the sequences for the B2M were kept confidential by Primerdesign Ltd.

qPCR reactions were run in a real time PCR thermo-cycler machine (Viia^TM^ 7 Real Time PCR System, ThermoFisher Scientific, Loughborough, UK) using the following conditions: 50 °C for 2 min and 95 °C for 10 min followed by 40 cycles of 95 °C for 15 s and 60 °C for 1 min. Gene expression was analyzed by the 2^∆∆*C*t^ method. Samples from at least six mice were used for each group and reactions for each sample were run in triplicate. 

### 4.8. Immunofluorescence Staining

Sagittal sections (7 µM) were cut from lung embedded at an optimal cutting temperature (OCT) compound using a cryostat (Cryostar^TM^ NX70 Cryostat from, Thermofisher Scientific). Sections were fixed in ice cold (−20 °C) methanol for 20 min and rehydrated in phosphate buffer saline (PBS, pH = 7.4). Sections were permeablised in PBS containing 0.1% (*v*/*v*) Triton-X100 before being blocked in 5% (*w*/*v*) skimmed milk in PBS solution for 30 min at room temperature. Sections were stained with primary antibody (rabbit polyclonal anti-Cx43, 1:100; Sigma-Aldrich Company Ltd., Gillingham, Dorset, UK) and incubated at 4 °C overnight. The next day, sections were washed in PBS for 30 min at room temperature and incubated with secondary antibody (goat anti-rabbit conjugated to Alexa flour 488, 1:500; Fisher Scientific) at 4 °C for 2 h. Nuclei were counter-stained with DAPI (1:1000; Thermofisher. Loughborough, UK). Mounting medium was applied on tissue sections and cover slides were applied. Slides were then examined under the LSM 800 Carl ZEISS confocal microscope, (Königsallee, Germany) for immunoreactivity.

### 4.9. Statistical Analysis

All data were shown as mean ± S.E.M. Data for cumulative concentration response curves (CRCs) were analyzed using GraphPad Prism 6 software (La Jolla, CA, USA). Data were fitted to a logistic equation, CRCs were generated, and EC_50_ values were derived. Global differences in CRCs were compared by two-way ANOVA with the Bonferroni’s post hoc test. Changes in the logarithm of median effective concentration (Log EC_50_), maximal contractile responses (E_max_), and maximal relaxation responses (R_max_) between two different groups were analyzed by using the Student’s *t*-test. 

## Figures and Tables

**Table 1:** The contractile effects of ET-1 and 5-HT in IPAs of male and female wildtype (WT) and *Cx43* heterozygous (*Cx43^+/−^*) mice.

Agonists	Groups	Log EC_50_ (M)	E_max_ (%)	Global Shift in Concentration Response Curve	*n*
ET-1	Male WT	−8.46 ± 0.08	131.8 ± 2.5	NS	6
	Male *Cx43^+/−^*	−8.77 ± 0.12 *	127.4 ± 2.8		5
ET-1	Female WT	−8.31 ± 0.06	126 ± 2.2	NS	5
	Female *Cx43^+/−^*	−8.48 ± 0.04	130.3 ± 4.5		7
5-HT	Male WT	−6.67 ± 0.05	112.7 ± 2.8	NS	6
	Male *Cx43^+/−^*	−6.64 ± 0.07	118.9 ± 4.8		5
5-HT	Female WT	−6.82 ± 0.05	119.6 ± 3.5	NS	5
	Female *Cx43^+/−^*	−6.83 ± 0.1	116.6 ± 6.7		5

Log EC_50_ indicates logarithm of median effective concentration. E_max_ maximal contractile effect. Global differences in concentration response curves were compared by two-way ANOVA. Changes in logarithm of median effective concentration (Log EC_50_) and maximal contractile responses (E_max_) between two different groups were analyzed by the Student’s *t*-test. NS: not significant. * *p* < 0.05 compared to male WT mice. Data are shown as mean ± S.E.M.

**Table 2:** Log EC_50_ (logarithm of median effective concentration) and R_max_ (maximal relaxation) values for MCh-induced or isoprenaline-induced relaxation.

Agonists	Groups	Log EC_50_ (M)	R_max_ (%)	Global Shift in Concentration Response Curve	*n*
MCh	Male WT	−6.93 ± 0.08	55.6 ± 6.8	****	5
	Male *Cx43^+/−^*	−7.14 ± 0.21 **	28 ± 4.1 **		5
MCh	Female WT	−6.52 ± 0.14	55.3 ± 8.5	****	6
	Female *Cx43^+/−^*	−6.48 ± 0.24	26.8 ± 7.8 *		6
MCh	Male WT	−6.39 ± 0.2	42.1 ± 4.6	****	6
	Plus ^37,43^Gap27	−6.08 ± 0.1 ****	22.9 ± 5.6 *		6
MCh	Female WT	−6.8 ± 0.08	63.1 ± 4.8	****	6
	Plus ^37,43^Gap27	−6.36 ± 0.1 **	42.2 ± 5.2 *		6
MCh	Male WT	−6.27 ± 0.11	32.8 ± 4.6	****	6
	Plus l-NAME	not applicable	2.2 ± 3.4 ***		6
Isoprenaline	Male WT	−7.77 ± 0.17	92.1 ± 1.19	****	5
	Plus ^37,43^Gap27	−7.41 ± 0.16	80.8 ± 4.2 *		5
Isoprenaline	Male WT	−7.84 ± 0.2	86.7 ± 3.2	****	6
	Plus l-NAME	−7.5 ± 0.13	60.4 ± 4.3 ***		6

Global differences in concentration response curves were compared by two-way ANOVA. Changes in logarithm of median effective concentration (Log EC_50_) and maximal relaxation responses (R_max_) between two different groups were analyzed by using the Student’s *t-*test. Data are shown as mean ± S.E.M. * *p* < 0.05, ** *p* < 0.01, *** *p* < 0.001 and **** *p* < 0.0001.

**Table 3:** The contractile effects of ET-1 and 5-HT in intra-lobar pulmonary arteries of hypoxic wildtype and *Cx43* heterozygous mice.

Agonists	Genotypes	Log EC_50_ (M)	E_max_ (%)	Global Shift in Concentration Response Curve	*n*
ET-1	Hypoxic WT	−7.95 ± 0.06	91.7 ± 8.1	**	6
	Hypoxic *Cx43^+/−^*	−8.07 ± 0.09 ***	112.7 ± 5.6 *		7
5-HT	Hypoxic WT	−6.56 ± 0.04	126 ± 2.2	NS	7
	Hypoxic *Cx43^+/−^*	−6.5 ± 0.03	130.3 ± 4.5		7

Log EC_50_ indicates the logarithm of median effective concentration, E_max_ maximal contractile effect. Global differences in concentration response curves were compared by using two-way ANOVA. Changes in logarithm of median effective concentration (Log EC_50_) and maximal contractile responses (E_max_) between two different groups were analyzed by using the Student’s *t-*test. NS: not significant * *p* < 0.05, ** *p* < 0.01, *** *p* < 0.001. Data are shown as mean ± S.E.M.

**Table 4:** TaqMan probe primers and their sequences for qPCR reactions.

Gene	Sequence	Product Length (Base Pairs)	Tm (°C)
*GJA1 * (Cx43)	Sense-ACTGAGCCCATCCAAAGACT Antisense-CAGGAGGAGACATAGGTGAGAG	95	Sense-56.6 Antisense-57.3
*GJA5 * (Cx40)	Sense-ATGGTATACTCTCCTCAGCACTAC Antisense-CCAGTCATTGAGAAGACTCAGAAC	117	Sense-56.8 Antisense-57
*GJA4 * (Cx37)	Sense-ACACCCACCCTGATCTACCT Antisense-TCCCTCTTTCTGCCGCAAC	75	Sense-57.5 Antisense-58
*GJC1 * (Cx45)	Sense-CAGAGATGGAGTTAGAAAGCGAAA Antisense-AAGCCCACCTCAAACACAGT	148	Sense-57.8 Antisense-57.7
*Panx1*	Sense-TCAGCCTCATTAACCTCATTGTG Antisense-TGGGCAGGATTTCATACACTTTG	114	Sense-57.5 Antisense-58
*NOS3 * (eNOS)	Sense-GGAAGTAGCCAATGCAGTGAA Antisense-GCCAGTCTCAGAGCCATACA	97	Sense-56.8 Antisense-57.2
*BMPR2 (BMPRII)*	Sense-TGTTATCAGTGACTTTGGTTTATCC Antisense-CTTATAGCCCGCATTATCTTCTTCC	84	Sense-56.5 Antisense-56.3
*Tph1*	Sense-AATTCACGGAAGAAGAGATTAAGAC Antisense-CCAGTTGCGGGATGTTGTC	150	Sense-56.3 Antisense-56.9
